# Playing tactics, contextual variables and offensive effectiveness in English Premier League soccer matches. A multilevel analysis

**DOI:** 10.1371/journal.pone.0226978

**Published:** 2020-02-18

**Authors:** Joaquín González-Rodenas, Rodrigo Aranda-Malaves, Andrés Tudela-Desantes, Félix Nieto, Ferran Usó, Rafael Aranda

**Affiliations:** 1 Department of Recreation and Sport Pedagogy, Ohio University, Columbus, Ohio, United States of America; 2 Department of Physical Education and Sports, University of Valencia, Valencia, Spain; 3 Doctoral School, Chatolic University San Vicente Mártir, Valencia, Spain; University of Cassino e Lazio Meridionale, ITALY

## Abstract

The aim of this study was to investigate the combined effects of tactical and contextual indicators on achieving offensive penetration and scoring opportunities in English Premier League (EPL) soccer matches. A total of 1971 team possessions from 20 random matches were evaluated by means of multidimensional observation. The EPL matches had a great proportion of fast attacks (36.0%) followed by combinative (29.6%), direct attacks (24.1%) and counterattacks (9.5%). Multilevel logistic regression models revealed that counterattacks (OR = 3.428; 95% CI: 2.004–5.864; P<0.001) were more effective to create goal scoring opportunities than combinative attacks, while direct attacks showed to be less effective (OR = 0.472; 95% CI: 0.264–0.845; P<0.05). Playing at home increased the probability (OR = 1.530; 95% CI: 1.097–2.135; P<0.05) of creating goal scoring opportunities compared with playing away. These findings show the multifactorial character of soccer and how different contextual and tactical indicators can influence the creation of offensive penetration and goal scoring opportunities in the English Premier League.

## Introduction

English Premier League (EPL) is one of the top sports competitions in the world and currently one of the best ranked soccer competitions in Europe according to the UEFA rankings. [1]This league possesses a strong history and soccer culture [2, 3] and compared to other domestic competitions, EPL seems to possess a more direct style of play [4], faster game tempo [5], higher number of heading and ground duels [6] and more aggressiveness [7] than other top European leagues. In recent years, this competition has evolved tactically and physically by increasing the number of passes, passing tempo, passing accuracy and high intensity running [8,9]. This evolution seems to be driven especially by the highest ranked clubs, who implement a more possession-based style compared to lower ranked clubs, who still play a more direct style [10].

However, although a great number of scientific studies about EPL have focused on physical demands [11, 12, 13] there is a lack of studies describing playing styles and their effectiveness to achieve offensive performance in this league [14]. In this sense, the technology currently available for clubs and sport scientists (e.g. global positioning systems (GPS), accelerometers, etc) allows them to track movements, impacts, speeds and distances and makes possible the optimal realization of valid and reliable studies on physical variables. Otherwise, the characterization of playing tactics may require a more complex process due to the dynamic, interactive and multifactorial nature of soccer actions, that depends on the interdependence between players skills, collective synergies, game plans, opponent behaviors and contextual variables [15].

For that purpose, a vast quantity of research studies have shown that systematic observation is a suitable methodology for analyzing tactical behaviors in sport [16], because it permits the inclusion of categorical data from the qualitative evaluation of different dimensions of match performance, and may improve the ability to describe soccer match play actions [17, 18]. In this sense, the use of multivariate logistic regression to study the combined and interactive effects of different tactical variables has been shown to be an appropriate method for the analysis of the offensive process in soccer in different competitions [19, 20, 21, 22, 23]. For the analysis of offensive performance, recent studies have suggested that tactical performance measures should not be directly related to the probability of goal scoring, but other dimensions related to creating space and disordering the opposing defense should be considered when analyzing styles of play in soccer [24, 25]. For that reason, in addition to goal and goal scoring opportunities, the analysis of successful penetration through defensive lines have been suggested as a novel dimension to study the offensive performance in recent studies [26, 27, 28].

Therefore, due to the lack of studies on the effectiveness of soccer styles of play in general, and in EPL in particular, the aim of this study was to investigate the effects of tactical and contextual indicators on achieving offensive penetration and scoring opportunities in English Premier League matches.

## Material and methods

### Sample

The unit of analysis was a “team possession” that started in open play. For the concept of team possession, the definition of Pollard and Reep [29, p.542] was used:

“*A team possession starts when a player gains possession of the ball by any means other than from a player of the same team. The player must have enough control over the ball to be able to have a deliberate influence on its subsequent direction. The team possession may continue with a series of passes between players of the same team but ends immediately when one of the following events occurs: a) the ball goes out of play; b) the ball touches a player of the opposing team (e.g. by means of a tackle, an intercepted pass or a shot being saved). A momentary touch that does not significantly change the direction of the ball is excluded*”.

Each match from EPL 2017–2018 season was assigned with a number from 1 to 380. An online random number generator [30] was used to select 20 random matches. The selected matches were downloaded from Wyscout platform (Wyscout Spa, Italy). This platform is a large private video database that allows soccer coaches, agents and analysts to watch and download all the matches from multiple leagues around the world. A total of 3620 team possessions were identified and analyzed. Those possessions where the duration was extremely short (0–3 seconds) and where it was not no possible to observe their playing tactics were excluded (n = 161; 4.5%). Finally, 1971 team possessions (54.4%) that started in an open play situation, were included in the study.

### Variables

The study used the REOFUT theoretical framework based on observational methodology and multidimensional analysis [31]. This instrument describes how to analyse multiple tactical and technical dimensions related to the start, development and the end of teams’ possessions, as well as their association with achieving offensive performance. This instrument has been used in multiple research studies to analyze different competitions and teams [22, 32] In this sense, this study includes the analysis of four independent tactical dimensions related to the possession start (initial penetration and initial defensive pressure) and possession development (duration of the attack and type of attack) (Table 1). Additionally, five independent contextual dimensions were analyzed (match location: “*home; away*”, match status: “*losing*, *drawing*, *winning*”, quality of opponent and quality of the observed team: “*high-ranked*: from first position to fifth position in the moment of the observed match; *medium-ranked*: from sixth position to fifteenth position in the moment of the observed match; *low-ranked*: from sixteenth position to twentieth position in the moment of the observed match”; and time of the match: “*first half; second half”*).

**Table 1:** Descriptions and definitions of tactical dimensions and categories (independent variables).

Variable	Definition	Categories
**Initial penetration**	Degree of offensive directness in the first three seconds of the team possession:	**1. Penetrative action:** passes or dribbles towards the opponent’s goal past opponent player (s) performed during the first three seconds of the ball possession [22].
		**2. Non-penetrative action:** any technical action towards any direction that does not past opponent player (s) performed during the first three seconds of the ball possession.
**Initial opponent pressure**	Distance between the player/s with the ball (first attackers) and the immediate pressing opponent player(s) (first defender(s)) during the first three seconds of the ball possession.	**1. Initial pressure:** one or several opponent players pressure the attackers within the first 3 seconds of the possession (the defender(s) are always located within 1.5 meters of the first attackers [22].
		**2. Non-initial pressure:** there are not any players that pressure the attacker (s) during the first 3 seconds of the possession.
**Duration of the attack**	Duration of the offensive sequence in seconds.	**1. Very short** (0–10 sec).
		**2. Short** (11–20 sec).
		**3. Long** (21–30 sec).
		**4. Very long** (31 or more seconds).
**Type of attack**	Degree of offensive directness [19, 20, 22, 23] in the offensive process.	**1. Combinative attack:** a)the possession starts by winning the ball in play or restarting the game, b) the progression towards the goal has a high number of non-penetrative and short passes, c)the circulation of the ball takes place more in width than in depth [23] and the intention of the team is to disorder the opponent using a high number of passes and slow tempo (Evaluated qualitatively), d) the opposing team has the opportunity to minimize surprise, reorganize his system and be prepared defensively.
		**2. Direct attack:** a) the possession starts by winning the ball in play or restarting the game, b) the progression towards the goal is based on one long pass from the defensive players to the forward players (evaluated qualitatively), c) the circulation of the ball takes place more in depth than in width and the intention of the team is to take the ball directly near the goal area to have opportunities of finishing by using a reduced number of passes and high tempo, d) the opposing team has the opportunity to minimize surprise, reorganize his system and be prepared defensively.
		**3. Fast attack:** a) the possession starts by winning the ball in play or restarting the game, b) the progression towards the goal has a high number of penetrative and short passes, c) the circulation of the ball takes place in width and depth [23] and the intention of the team is to disorder the opponent with a reduced number of passes and high tempo (evaluated qualitatively), d) the opposing team has the opportunity to minimize surprise, reorganize his system and be prepared defensively.
		**4. Counterattack:** a) the possession starts by winning the ball in play, b) the progression towards the goal attempts to utilize a degree of imbalance right from start to the end with high tempo [19], c) the circulation of the ball takes place more in depth than in width and the intention of the team is to exploit the space left by the opponent when they were attacking, d) the opposing team does not have the opportunity to minimize surprise, reorganize his system and be prepared defensively.

**Fig 1:**
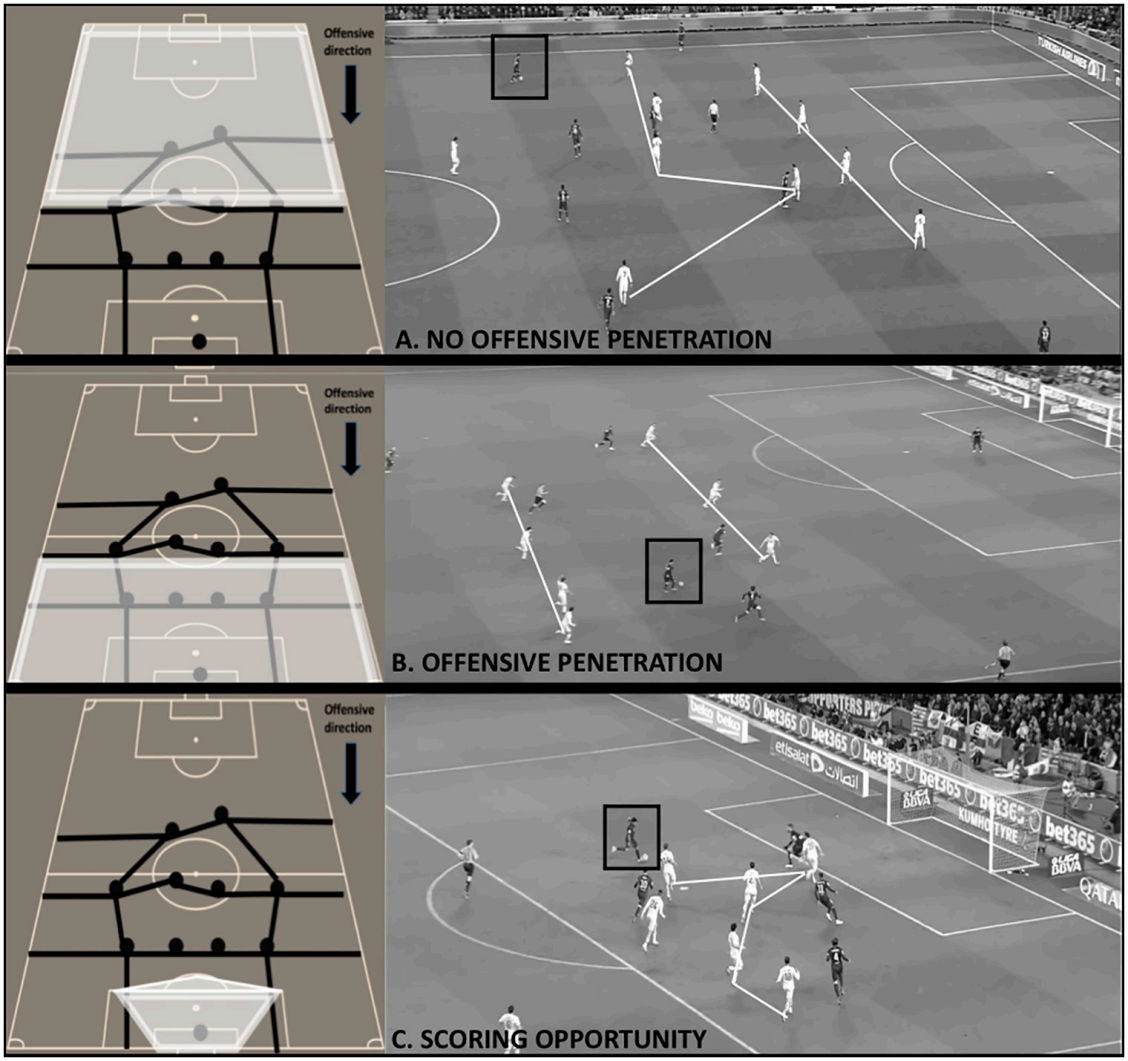
Graphical representation and real example of the three possible offensive outcomes. **A) No offensive penetration:** The team possession does not achieve to disorder and beat the forwards or midfielders’ lines of the opposing team during the offensive sequence. **B) Offensive penetration:** The team possession achieves to beat the forwards and midfielders’ lines of the opponent and face directly the defensive line during the offensive sequence but the possession ends without creating any scoring opportunity. The player(s) facing the defensive line has/have enough time and space to perform intended actions on the ball at the moment of receiving the ball. C) **Scoring opportunity:** The team has a clear chance of scoring a goal during the ball possession. This includes all goals, all shots produced inside the score pentagon*, those shots produced outside the score pentagon that pass near the goal (evaluated qualitatively) and all chances of shooting inside the score pentagon (the player is facing the goal, there are not any opponents between him and the goal and he has enough space and time to make a playing decision). * Score pentagon is used as the zone of reference because it selects the space with high shooting angle and a short distance to goal (20 meters or less) which are very important factors to achieve goals [33, 34].

For the possession outcome, the dependent variable “offensive performance” was evaluated. This variable analyzes the degree of penetration over the opposing defense and the creation of goal scoring opportunities during the team possession. This variable has three categories that are defined in Fig 1 (1. *No offensive penetration*; 2: *offensive penetration* and 3: *scoring opportunity*).

For obtaining a more detailed analysis of the different degrees of offensive performance, this study grouped the categories in two different ways in order to create two possible dependent variables with two categories. On one hand, we grouped “1. *no offensive penetration*” and “2, *offensive penetration*” to create the category “*no scoring opportunity*” as a part of the first dependent variable named “scoring opportunity”. On the other hand, we grouped “2. *offensive penetration*” and “3. *scoring opportunity*” to create the category “*high penetration*” as a part of the second dependent variable named “offensive penetration”. This organization of the categories allows us to study two different offensive outcomes based on penetration and scoring opportunities.

### Match performance analysis

The study is based on the principles of observational methodology [16]. For the analysis, a researcher with experience in match analysis and soccer coaching completed a theoretical and practical training on the use of the REOFUT instrument. The software Lince [35] was used to observe the matches, code the variables and categories and register the data. Inter-observer and intra-observer analysis showed appropriate levels of reliability for the tactical variables analysed in the study based on Cohen’s Kappa calculations after the analysis of 107 team possessions (initial penetration: 0.819, 0.963; initial defensive pressure: 0.815, 0.816; duration of the attack: 0.958, 0.963; type of attack: 0.776, 0.898, for inter and intra-observer reliability, respectively).

### Statistical analysis

All the analyses were performed using SPSS software (IBM SPSS, Version 20.0). An analysis of frequencies was carried out to describe the characteristics of the sample and the occurrence of each tactical dimension according to the offensive performance.

**Fig 2:**
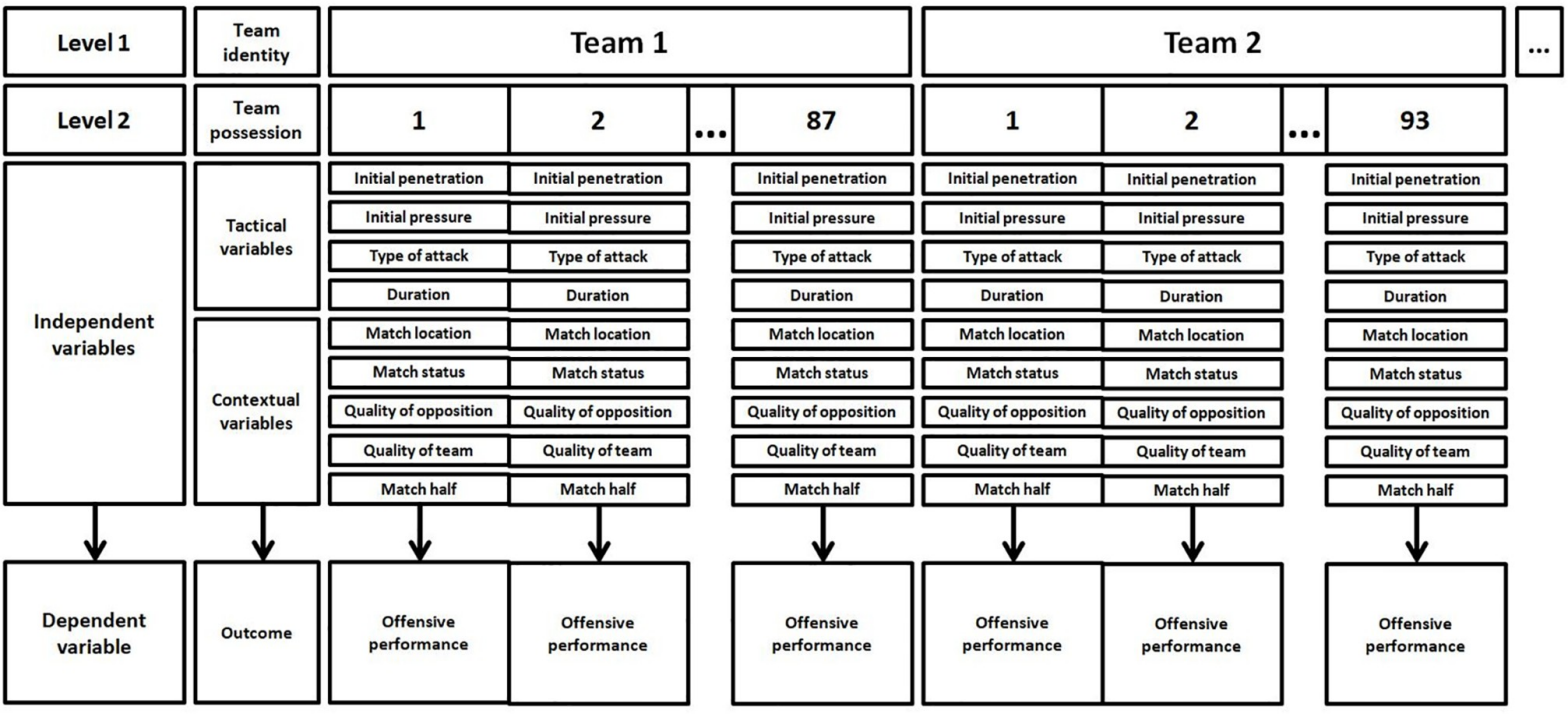
Hierarchical data structure, in which team possessions are nested in teams.

Due to the hierarchal structure of ball possessions in soccer (each team has its own tactical style and different ways to achieve offensive effectiveness), multilevel modeling [36]was carried out to cluster the ball possessions (Level 2) within teams (Level 1) (Fig 2). A mixed model was created to analyze the effect of the contextual (match location, quality of opposition, match status, quality of the team and match half) and tactical (initial penetration, initial opponent pressure, duration of the attack and type of attack) independent variables (fixed effects). on the creation of offensive performance, considering the team identity (random effects).

With this organization of the data, binary logistic regressions were constructed to predict the outcome related to offensive performance: offensive penetration (0 = No offensive penetration, 1 = high offensive penetration) and scoring opportunity (0 = no scoring opportunity, 1 = scoring opportunity).

Firstly, unadjusted models (univariate analysis) were carried out to determine the association of each independent variable with the dependent variable. Secondly, based on the unadjusted models described above, adjusted logistic multilevel models (Multivariate analysis) were constructed with all significant independent variables from the unadjusted models included.

## Results

### Descriptive analysis

Table 2 shows the main characteristics of the sample. EPL matches had a great proportion of fast attacks (36.0%) followed by combinative (29.6%) and direct attacks (24.1%) while only 9.5% progressed as a counterattack. The team possessions had a high frequency of “initial pressure” (74.3%), very short or short duration (50.7% or 26.1%, respectively) and more proportion of non-penetrative actions at the beginning of the possession (56.9%).

**Table 2:** Descriptive characteristics of the sample.

Variable	N	No offensive penetration	Offensive penetration	Scoring Opportunity
		N (%)	N (%)	N (%)
**Initial penetration**				
No penetration	1115 (56.9)	633 (56.8)	421 (37.8)	61 (5.5)
Penetration	844 (43.1)	269 (31.9)	470 (55.7)	105 (12.4)
**Initial opponent pressure**				
Initial Pressure	1382 (74.3)	669 (48.4)	602 (43.6)	111 (8.0)
Non-Initial Pressure	479 (25.7)	162 (33.8)	270 (56.4)	47 (9.8)
**Duration of the attack**				
Very short (0–10 sec)	994 (50.7)	613 (61.7)	293 (29.5)	88 (8.9)
Short (11–20 sec)	512 (26.1)	175 (34.2)	298 (58.2)	39 (7.6)
Long (21–30 sec)	262 (13.3)	84 (32.1)	159 (60.7)	19 (7.3)
Very long (31+ sec)	190 (9.7)	30 (15.8)	142 (74.7)	18 (9.5)
**Type of attack**				
Combinative attack	535 (29.6)	182 (34.0)	313 (58.5)	40 (7.5)
Direct attack	450 (24.1)	346 (76.9)	97 (21.6)	7 (1.6)
Counterattack	173 (9.5)	33 (19.10)	97 (56.1)	43 (24.9)
Fast attack	652 (36.0)	218 (33.4)	367 (56.3)	67 (10.3)
**Match Location**				
Away	895 (45.6)	460 (51.4)	380 (42.5)	55 (6.1)
Home	1068 (54.4)	445 (41.7)	512 (47.9)	111 (10.4)
**Quality of Opposition**				
Low-ranked	344 (18.3)	135 (39.2)	186 (54.1)	23 (6.7)
Medium-ranked	1116 (59.5)	502 (45.0)	502 (45.0)	112 (10.0)
High-ranked	415 (22.2)	223 (53.7)	166 (40.0)	26 (6.3)
**Match Status**				
Losing	452 (23.0)	194 (42.9)	222 (49.1)	36 (8.0)
Drawing	952 (48.5)	464 (48.7)	423 (44.4)	65 (6.8)
Winning	559 (28.5)	247 (44.2)	247 (44.2)	65 (11.6)
**Quality of Team**				
Low-ranked	353 (18.8)	176 (49.9)	146 (41.4)	31 (8.8)
Medium-ranked	1017 (54.2)	514 (50.5)	424 (41.7)	79 (7.8)
High-ranked	505 (27.0)	170 (33.7)	284 (56.2)	51 (10.1)
**Match Half**				
First	1005 (51.2)	486 (48.4)	441 (43.9)	78 (7.8)
Second	956 (48.8)	416 (43.5)	452 (47.3)	88 (9.2)
**Total**	1971	902 (46.0)	891 (45.5)	166 (8.5)

As far as offensive performance, the 45.5% of team possessions achieved offensive penetration, while the 8.5% created a scoring opportunity.

### Multilevel logistic regression analysis

Table 3 shows how in the baseline model, no significant differences were found in the odds of achieving high offensive penetration versus no offensive penetration. On the other hand, teams had lower odds of creating goal scoring opportunities during ball possession, in comparison with non-creating a goal scoring opportunity.

**Table 3:** Baseline model (Intercept) for the prediction of high penetration vs no penetration and scoring opportunity vs no scoring opportunity.

Offensive Performance						95% CI	
	Coefficient	Std. Error	t	Sig	Exp	Lower	Upper
**High penetration**	0.093	0.130	0.712	0.476	1.097	0.850	1.417
**Scoring Opportunity**	-2.404	0.098	-24.603	0.000	0.090	0.075	0.109

Regarding the random effects, Table 4 shows how the effect of ‘team identity’ did not present a significant variance for the creation of goal scoring opportunities but this effect was significant for the achievement of high offensive penetration.

**Table 4:** Random effects of team identity on achieving high penetration vs no offensive penetration and scoring opportunity vs no scoring opportunity.

Offensive performance					95% CI	
	Estimate	Std. Error	Z	Sig	Lower	Upper
**Scoring opportunity**	0.046	0.052	0.882	0.378	0.005	0.422
**High penetration**	0.259	0.108	2.396	0.017	0.114	0.588

For offensive tactics, Table 5 shows how both univariate and multivariate analysis showed that ‘Initial penetration’, ‘non-initial pressure’, ‘short, long and very long possessions’ and ‘counterattacks’ obtained higher probabilities to achieve offensive penetration than ‘non-penetration’, ‘initial pressure’, ‘very short possessions’ and ‘combinative attacks’, respectively. Also, ‘combinative attacks’ were more effective to achieve offensive performance than ‘direct attacks.

**Table 5:** Multilevel binary logistic regression predicting to achieve high penetration vs low penetration (reference category).

Variable	High penetration vs low penetration (univariate Analysis)			High penetration vs low penetration (multivariate analysis)		
	β	SE	OR (95% CI)	β	SE	OR (95% CI)
**Initial penetration**						
No penetration (Ref)						
Penetration	1.048	0.099	2.851 (2.348–3.462)***	0.670	0.150	1.954 (1.458–2.620)***
**Initial pressure**						
Initial pressure (Ref)						
Non-initial pressure	0.573	0.115	1.773 (1.415–2.222)***	0.448	0.149	1.565 (1.169–2.095)**
**Duration of the attack**						
Very short (0–10) (Ref)						
Short (11–20)	1.124	0.118	3.078 (2.444–3.876)***	1.499	0.162	4.477 (3.261–6.146)***
Long (21–30)	1.280	0.152	3.596 (2.669–4,846)***	1.789	0.214	5.986 (3.936–9.102)***
Very long (31+)	2.094	0.213	8.116 (5.340–12.335)***	2.755	0.278	15.718 (9.118–27.096)***
**Type of attack**						
Combinative (Ref)						
Direct attack	-1.848	0.152	0.158 (0.117–0.212)***	-0.928	0.193	0.396 (0.271–0.577)***
Fast attack	0.041	0.221	1.042 (0.810–1.339)	0.965	0.192	2.625 (1.802–3.822)***
Counterattack	0.856	0.128	2.353 (1.525–3.631)***	2.193	0.298	8.960 (4.998–16.063)***
**Match Location**						
Away (Ref)						
Home	0.453	0.102	1.573 (1.287–1.922)***	0.530	0.163	1.700 (1.234–2.341)**
**Quality of Opposition**						
Low-ranked (Ref)						
Medium-ranked	-0.579	0.159	0.561 (0.410–0.766)***	-0.615	0.256	0.541 (0.327–0.894)*
High-ranked	-0.599	0.197	0.550 (0.373–0.809)**	-0.829	0.281	0.436 (0.251–0.758)**
**Match Status**						
Losing (Ref)						
Drawing	-0.227	0.130	0.797 (0.618–1.029)	-0.305	0.174	0.737 (0.524–1.036)
Winning	-0.306	0.155	0.736 (0.544–0.997)*	-0.472	0.225	0.624 (0.402–0.970)*
**Quality of Team**						
Low-ranked (Ref)						
Medium-ranked	-1.314	0.242	0.269 (0.167–0.432)**	-0.233	0.338	0.792 (0.408–1.538)
High-ranked	-0.432	0.261	0.649 (0.389–1.084)	0.254	0.345	1.289 (0.655–2535)
**Match Half**						
First (Ref)						
Second	0.210	0.093	1.234 (1.027–1.481)*	0.172	0.125	0.188(0.930–1.518)
**Intercept**	0.093	0.130	1.097 (0.850–1.417)	-0.907	0.386	0.404 (0.190–0.861)*

β = *Coefficient; SE = Standard error; OR = Odds Ratio; CI = Confidence interval for odds ratio;*

* = p<0.05

** = p<0.01

*** = P<0.001

For contextual variables, both univariate and multivariate analysis indicated that “playing at home”, playing against a “low-ranked opponent”, and the moment of ‘losing’ the game were more effective to achieve offensive penetration in comparison with ‘playing away’, playing against a ‘medium or high-ranked opponent’ and during the moment of ‘winning the game’, respectively.

**Fig 3:**
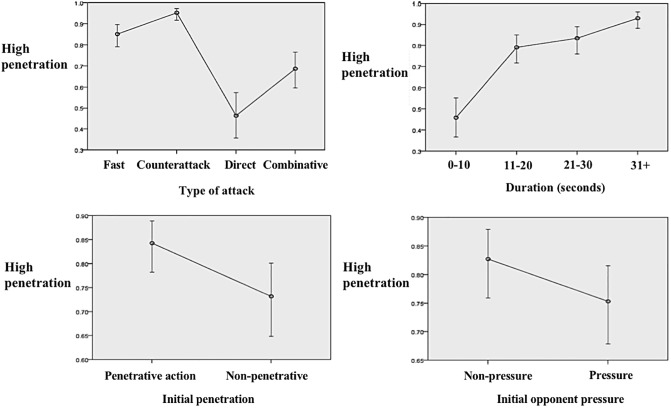
Predicted probabilities to create offensive penetration according to different tactical dimensions after adjusting for the variables included in the multivariate analysis.

Fig 3 shows how, after controlling for the rest of the variables, counterattacks and fast attacks showed more than 85% of probabilities to achieve offensive penetration, whereas direct attacks obtained less than 50%. In terms of the duration of the attack, the more elaboration and duration, the more offensive penetration was achieved.

Regarding the possession start, performing a penetrative action or starting the sequences without the opponent’s pressure obtained closer to 85% of probabilities to achieve offensive penetration.

Table 6 shows how for univariate analysis, only the tactical variables ‘initial penetration’ and ‘type of attack’ obtained differences in the odds of creating scoring opportunities depending on each category. For the multivariate analysis, only the type of attack had significant differences between tactics. In this way, counterattacks were more effective than combinate attacks to create scoring opportunities (OR = 3.428; 95% CI: 2.004–5.864; p<0.001), while directs attacks were less likely to produce a goal scoring opportunity than combinative attacks (OR = 0.472; 95% CI: 0.264–0.845; P<0.05). No differences were found between fast and combinative attacks.

**Table 6:** Multilevel binary logistic regression predicting to achieve scoring opportunity vs no scoring opportunity (reference category).

Variable	Scoring opportunity vs no scoring opportunity (univariate analysis)			Scoring opportunity vs no scoring opportunity (multivariate analysis)		
	β	SE	OR (95% CI)	β	SE	OR (95% CI)
**Initial penetration**						
No penetration (Ref)						
Penetration	0.893	0.169	2.443 (1.755–3.400)***	0.184	0.203	1.202 (0.807–1.790)
**Initial pressure**						
Initial pressure (Ref)						
Non-initial pressure	0.188	0.180	1.207 (0.848–1.719)			
**Duration of the attack**						
Very short (0–10) (Ref)						
Short (11–20)	-0.175	0.201	0.839 (0.565–1.246)			
Long (21–30)	-0.225	0.264	0.799 (0.476–1.340)			
Very long (31+)	0.039	0.273	1.040 (0.608–1.777)			
**Type of attack**						
Combinative (Ref)						
Direct attack	-0.810	0.296	0.445 (0.249–0.795)**	-0.751	0.297	0.472 (0.264–0.845)*
Fast attack	0.297	0.204	1.346 (0.901–2.010)	0.214	0.221	1.239 (0.803–1.812)
Counterattack	1.342	0.237	3.825 (2.401–6.092)***	1.232	0.274	3.428 (2.004–5.864)***
**Match Location**						
Away (Ref)						
Home	0.568	0.174	1.765 (1.254–2.483)**	0.425	0.170	1.530 (1.097–2.135)*
**Quality of Opposition**						
Low-ranked (Ref)						
Medium-ranked	0.441	0.249	1.554 (0.954–2.532)			
High-ranked	-0.067	0.310	0.935 (0.509–1.719)			
**Match Status**						
Losing (Ref)						
Drawing	-0.131	0.210	0.877 (0.581–1.324)			
Winning	0.370	0.215	1.448 (0.951–2.206)			
**Quality of Team**						
Low-ranked (Ref)						
Medium-ranked	-0.175	0.256	0.840 (0.508–1.388)			
High-ranked	-0.033	0.283	0.967 (0.555–1.685)			
**Match Half**						
First (Ref)						
Second	0.191	0.163	1.210 (0.879–1.665)			
**Intercept**	-2.404	0.098	0.090 (0.075–0.109)	-2742	0.202	0.064 (0.043–0.096)***

β = *Coefficient; SE = Standard error; OR = Odds Ratio; CI = Confidence interval for odds ratio;*

* = p<0.05

** = p<0.01

*** = P<0.001

For contextual variables, only the variable ‘Match location” showed how playing at home increased the odds of creating goal scoring opportunities in comparison with playing away OR = 1.530; 95% CI: 1.097–2.135; P<0.05).

**Fig 4:**
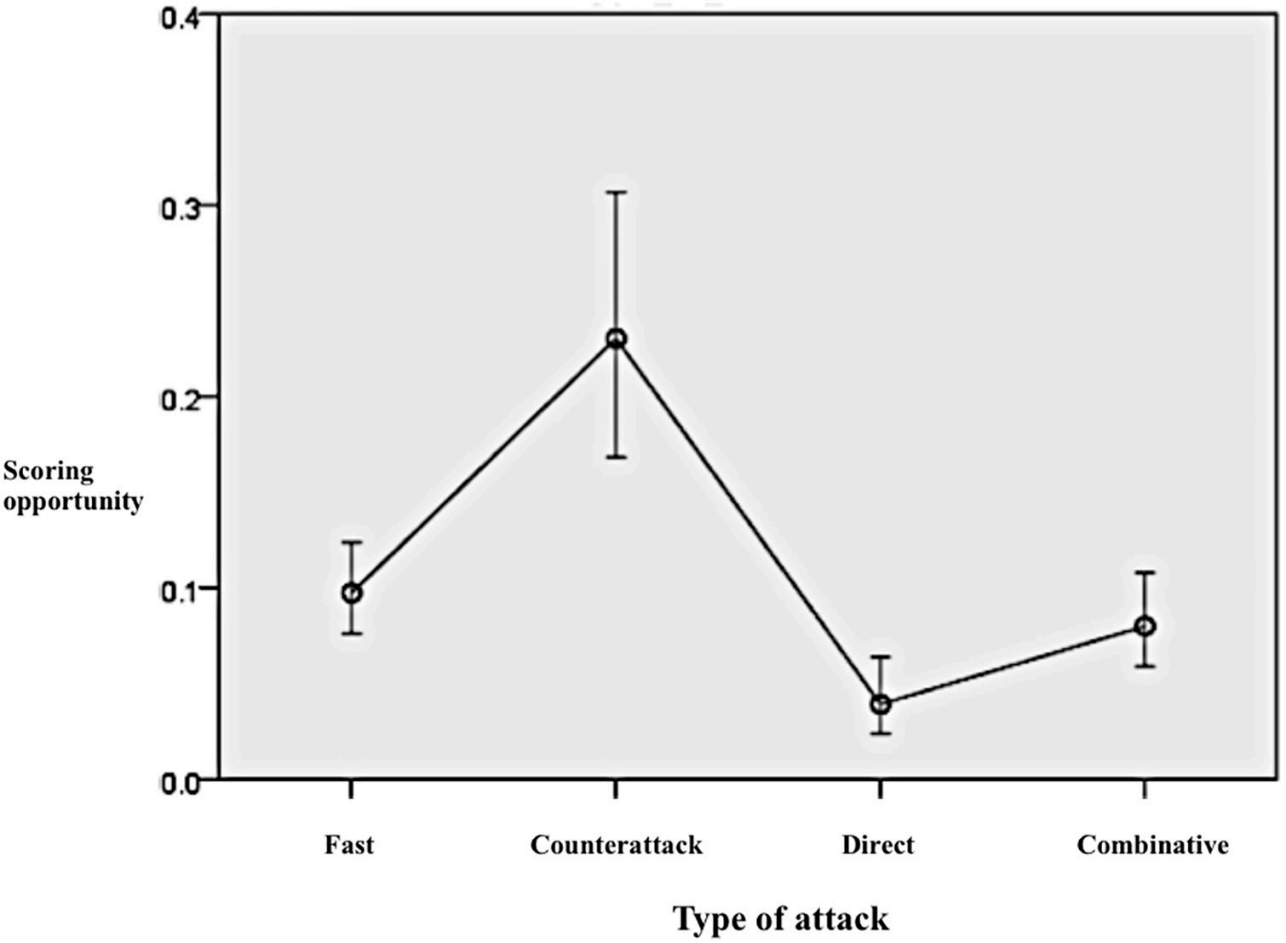
Predicted probabilities to create a scoring opportunity according to the type of attack after adjusting for the variables included in the multivariate analysis.

Fig 4 shows how, after controlling for the significant variables, counterattacks showed more than 20% of odds of creating scoring opportunities, while the direct attacks were the least effective offensive strategy, with less than 5% of probabilities.

### Discussion

The aim of this study was to investigate the combined effects of tactical and contextual indicators on achieving offensive penetration and scoring opportunities in English Premier League matches during the 2017–2018 season.

First of all, this study described some tactical characteristics from EPL matches. In this regard, 3 out of 4 team possessions started against defensive pressure and 43.1% tried to penetrate over the opponent during the first three seconds. For the possession development, fast and direct attacks comprised nearly 60% of the offensive sequences and 3 out of 4 attacks lasted less than 20 seconds. These results agree with previous studies that highlighted the fast game tempo and direct style of play of EPL [4,5]. These findings suggest that, although this competition has evolved towards a more combinative and passing style in recent years [8], there are still a great proportion of fast and direct attacks. The fact of describing the main tactical characteristics of the competition, makes very interesting the purpose of knowing what tactical dimensions are more effective than others.

For the possession start, our data revealed how performing initial penetration and the lack of defensive pressure in the first three seconds of the possession obtained higher probabilities for teams to achieve offensive penetration, although no differences were found for the creation of goal scoring opportunities when adjusting for the rest of significant variables. These results do not agree with the study of Gonzalez-Rodenas et al. [22] in Major League Soccer, Hughes and Lovell [37] in UEFA Champions League and Casal et al. [21] in the Eurocup 2008, where the initial penetration was key to create scoring opportunities. The specific tactical particularities of each competition may explain the differences between the research studies.

Regarding the possession development, univariate and multivariate analysis showed how counterattacks were more effective than combinative attacks both for achieving offensive penetration and creating goal scoring opportunities. This finding agrees with previous research in different competitions [19, 20, 22, 23] and it is not surprising due to the fact that counterattacks aim to exploit imbalances in the opponent’s defense. On the other hand, our study found how fast attacks were more effective than combinative attacks to achieve offensive penetration but not to create scoring opportunities, whereas direct attacks were less effective than combinative attacks for achieving offensive performance.

According to our knowledge, this is the first study that has compared the effectiveness of four types of attack, differentiating ‘fast’, ‘combinative’ and ‘direct’ within the positional moment, and ‘counterattack’ within the transitional moment of the game. For that reason, differences between research designs and the definition of “offensive success” make it difficult to compare our findings with other studies. For instance, Sarmento et al. [23] found that fast attacks increased the success of an offensive sequence by 40% compared with positional attacks in top teams from European soccer leagues and UEFA Champions League, but their definition of “success” not only included scoring opportunities but also the generation of direct free kicks, penalties and corner kicks.

As far as the duration of the attack, our study found how short, long and very long possessions were more effective than very short possessions to produce offensive penetration, but this variable did not show differences in the probability of creating scoring opportunities. In this sense, the fact of performing longer passing sequences would secure the penetration through the first two lines of the opponent and progress until dangerous zones, but this fact would not increase the odds of creating shooting situations. Previous literature has shown contradictory results. On one hand, several studies have found how longer duration produced more offensive effectiveness in the Norwegian [19] Spanish [20] and American [22] domestic leagues. On the other hand, Sarmento et al. [23] reported that increasing the possession duration by a single second in top European leagues, resulted in a decrease of 2% in the probability of success of the offensive sequence.

According to these findings, EPL has a predictable process to achieve offensive penetration, but a generally unpredictable way to create goal scoring opportunities, highlighting only the counterattacks as the most effective way of attacking. Therefore, these finding may help keep open the debate about whether long vs short or fast vs positional possessions are more effective to create scoring opportunities in this competition. This debate has been open since the pioneer study of Reep and Benjamin [38] that found how more than 80% of goals were scored from sequences of 3 or fewer passes. These findings influenced the playing styles of English teams and led to a direct style of play [2] based on moving the ball into a shooting position as directly as possible with the least number of passes. However, our study has found that despite the high amount of direct and fast attacks registered in this sample of EPL, these two types of attack were not more effective than counterattacks or combinative attacks to create scoring opportunities, and particularly direct attacks demonstrated to have extremely low effectiveness.

Regarding the contextual variables, our results support the fact that match status, quality of the opponent and match location influence the offensive penetration in EPL, although no great influence has been shown regarding the creation of goal scoring opportunities. In term of match status, our study found how losing teams obtained higher odds of achieving offensive penetration than winning teams. Previous literature suggests that, losing teams increase the build-up situations [39] have longer passing sequences [40] and increase ball possession [41]. This increase in offensive indicators may be due to the necessity of losing teams to score a goal in order to equalize the game, as well as the convenience of the winning teams in retaining the advantage achieved by means of implementing a more defensive behavior.

As for the quality of the opponent, playing against a “medium or low ranked” team increased the probabilities of penetrating offensively but not of creating goal scoring opportunities. In the line of our results, previous studies have found how playing versus weaker opposition was associated with more advanced recovery location on the field [42, 43], higher offensive length, width and surface area [44] and more time spent in possession [45].

For match location, univariate and multivariate analysis showed how “playing at home” obtained higher odds of achieving offensive penetration and scoring opportunities in comparison with ‘playing away’. The home advantage phenomena has been widely studied in the literature. The general scientific evidence supports the fact that teams implement a more offensive style of play and achieve more goals [46] and more effectiveness to create scoring opportunities [22] when playing at home rather than away. The study of Staufenbiel, Lobinger and Strauss [47] found how coaches had greater winning expectative, established more ambitious objectives and a more offensive strategy when playing at home. This fact may be related to the higher offensive deployment in terms of more shots, dribbles and passes [48], as well as more complex and structured attacking patterns [49].

To the best of our knowledge, this is the first study to explore the combined effects of contextual and tactical dimensions on the offensive performance exclusively in the English Premier League. Therefore, these results provide great information for coaches and performance analysts in order to reflect on the predominant styles of play in this competition and their association with offensive effectiveness. Regarding the limitations of our study, these results only reflect the playing style in this particular competition and they cannot be extrapolated to other soccer contexts. Also, we are aware of the limitations of observational methodology to capture the high tactical complexity of styles of plays in soccer, where the interaction between teammates, opponents and contextual variables create unique and variable situations in each team possession.

In conclusion, English Premier League showed a predictable process to achieve offensive penetration based on contextual and tactical factors, but only the type of attack and the match location showed differences between categories for the creation of scoring chances. For the creation of goal scoring opportunities, counterattacks were more effective and direct attacks were less effective than combinative attacks, whereas playing at home obtained higher effectiveness than playing away.

## Supporting information

S1 File EPL_data_base. (SAV)
